# Thyroid Storm in a Twin Pregnancy Presenting as Severe Hypertension and Preterm Labor

**DOI:** 10.1055/a-2788-1788

**Published:** 2026-01-29

**Authors:** Adwoa A. Baffoe-Bonnie, Lillian J. Dubiel, Lillian B. Boettcher, Maria Martinez Cruz, Amanda M. Craig

**Affiliations:** 1 Duke University School of Medicine, Durham, North Carolina, United States; 2 Department of Obstetrics and Gynecology, Duke University School of Medicine, Durham, North Carolina, United States; 3 Division of Maternal-Fetal Medicine, Department of Obstetrics and Gynecology, Duke University School of Medicine, Durham, North Carolina, United States; 4 Division of Endocrinology, Department of Internal Medicine, Duke University School of Medicine, Durham, North Carolina, United States; 5 Division of Maternal-Fetal Medicine, Department of Obstetrics and Gynecology, Duke University School of Medicine, Durham, North Carolina, United States

**Keywords:** thyroid storm, Graves' disease, thyrotoxicosis, twin pregnancy, preterm delivery

## Abstract

**Background:**

Thyroid storm is a rare, life-threatening endocrine emergency in pregnancy with significant maternal and neonatal implications. Clinical symptoms may mimic those of hypertensive disorders in pregnancy. Prompt diagnosis and multidisciplinary management are critical for reducing maternal and neonatal morbidity and mortality.

**Case:**

A 31-year-old woman with a dichorionic diamniotic twin pregnancy, chronic hypertension, short cervix, and poorly controlled Graves' disease presented at 22 weeks' gestation with vaginal bleeding and severe-range blood pressures. She was diagnosed with thyroid storm and admitted to the intensive care unit. She failed to respond to maximal medical management, ultimately requiring plasmapheresis. She later developed preterm labor and spontaneously delivered periviable twins. She eventually underwent total thyroidectomy for definitive management of thyroid storm.

**Conclusion:**

This case illustrates the severe consequences of poorly controlled Graves' disease in pregnancy. Although thyroid storm can mimic preeclampsia, abnormal thyroid function tests, persistent tachycardia, fevers, and hypertension with wide pulse pressures may help distinguish it. Timely recognition and multidisciplinary care are imperative to decrease morbidity and mortality associated with thyroid storm.

## Introduction

Thyrotoxicosis, the clinical state of excessive circulating thyroid hormone, is relatively uncommon in pregnancy, with a prevalence of 0.2 to 0.9%, most commonly due to Graves' disease or human chorionic gonadotropin-mediated gestational transient thyrotoxicosis. Among these cases, only 1 to 2% progress to thyroid storm, which can be precipitated by stressors such as surgery, trauma, delivery, or sepsis. 1 Thyroid storm is a life-threatening form of thyrotoxicosis characterized by multiorgan dysfunction, with reported maternal mortality of 20 to 30% in pregnancy. Thyroid storm risk in pregnancy increases in multifetal gestations and with uncontrolled baseline hyperthyroidism. 2 The diagnosis is clinical, as thyroid hormone lab derangements do not directly correlate with the development or severity of the condition. 3 Diagnostic scales, such as the Burch–Wartofsky Point Scale, can aid in evaluating thyroid storm, but should always be used in conjunction with clinical judgement, as scoring systems lack high sensitivity and specificity. 4

This is a case of thyroid storm in a second-trimester twin pregnancy with poorly controlled Graves' disease, complicated by severe hypertension and preterm labor. The patient did not respond to conventional thyroid storm medical therapy and ultimately required definitive treatment with total thyroidectomy.

### Case

This patient is a 31-year-old woman, gravida 3 para 2002, with two prior spontaneous vaginal deliveries, who presented to our institution at 22 ^2/7^ weeks of gestation for resolved postcoital vaginal bleeding that had occurred 2 days prior. This pregnancy was complicated by a dichorionic diamniotic twin gestation. She was diagnosed with a short cervix via transvaginal ultrasound at 19 ^5/7^ weeks of gestation with transvaginal cervical length measuring 2.1 cm. She was prescribed vaginal progesterone but had not yet initiated therapy at the time of presentation. Her pregnancy was otherwise complicated by chronic hypertension (no medications) and uncontrolled Graves' disease, which was diagnosed 3 months prior to pregnancy. The patient demonstrated poor methimazole (MMI) adherence at this time due to medication side effects. At diagnosis, thyroid-stimulating immunoglobulin was 29.30 IU/L (reference range [RR] < 0.55 IU/L) and thyroid receptor antibodies were 36.50 (RR < 1.75 IU/L).

During the first trimester, the patient was switched to propylthiouracil (PTU) 200 mg three times daily. The patient had two mild-range blood pressures (140s–150s/90s–100s mmHg) recorded at her initial prenatal visit (9 ^0/7^ weeks), which were attributed to missed doses of oral labetalol for chronic hypertension. After this visit, she self-discontinued PTU due to nausea.

She was transitioned to MMI 20 mg daily during the second trimester, but again self-discontinued it. During the second trimester, thyroid-stimulating hormone (TSH) remained suppressed (<0.01 mUI/mL; RR 0.34–5.66 µIU/mL).

In triage, her vitals were notable for elevated systolic blood pressures in the 150s mmHg (peak 161 mmHg) and tachycardia up to 109 bpm. She was afebrile with an oxygen saturation of 99% on room air. Physical exam revealed a slight tremor, but she was otherwise stable. Preeclampsia workup showed normal hematologic, renal, and hepatic function; urine protein-to-creatinine ratio was elevated at > 500. Given the recent vaginal bleeding, this abnormal value was suspected to be secondary to contamination; however, a new diagnosis of superimposed preeclampsia was also considered, and she was admitted for blood pressure and laboratory monitoring.

**Fig. 1:**
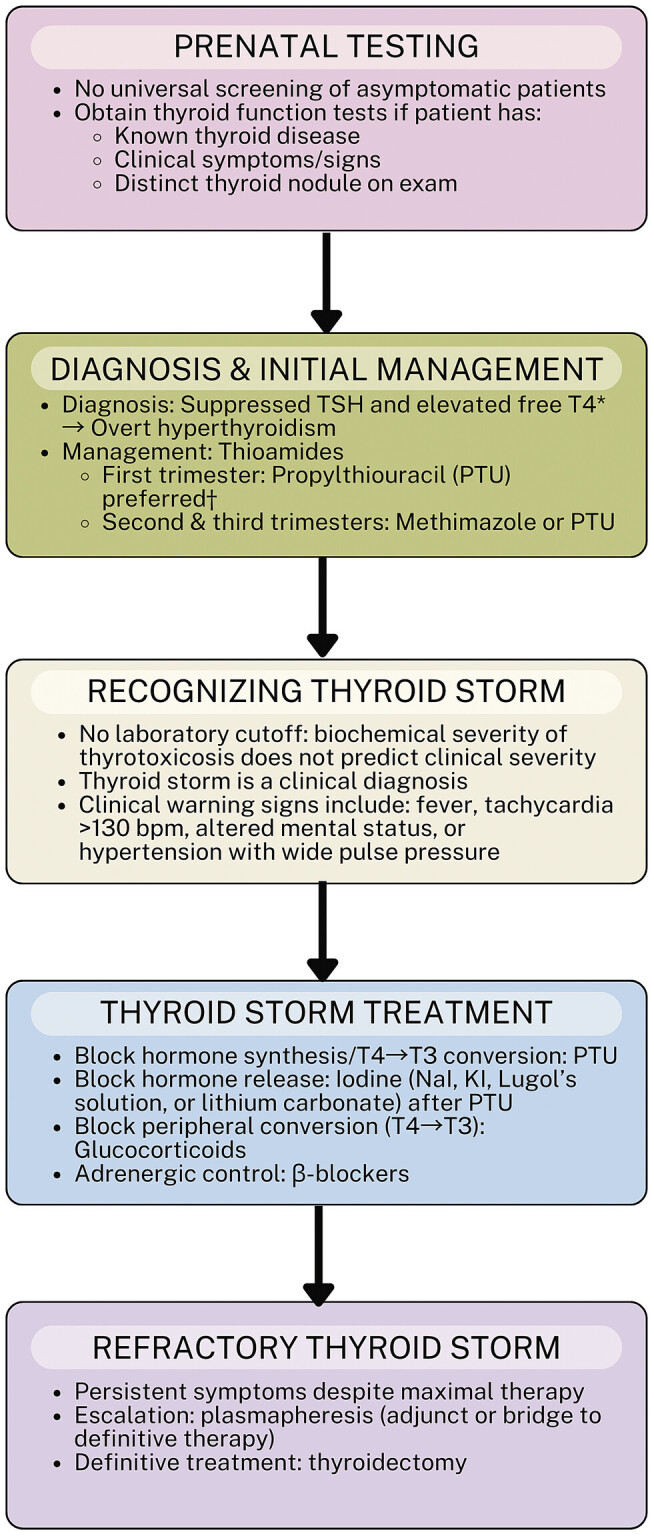
Management of thyroid disease and thyroid storm in pregnancy. Flowchart summarizing the approach to thyroid disease in pregnancy, including prenatal testing, hyperthyroidism diagnosis and initial management, recognition of thyroid storm, treatment strategies, and escalation to refractory interventions such as plasmapheresis or thyroidectomy. Key points highlight testing indications, first-line and alternative therapies, and clinical features of thyroid storm.

On hospital day 2, a repeat protein-to-creatinine ratio from a catheterized specimen returned within normal limits. Additional laboratory findings showed severely deranged thyroid function tests (free thyroxine: 3.94 ng/dL; RR: 0.52–1.21 ng/dL, free T3: 15.98 pg/mL; RR 2.40–4.12 pg/mL, TSH < 0.01 µIU/mL). MMI was restarted per endocrinology. The patient endorsed new abdominal cramping. Sterile vaginal exam showed 1 cm dilation, 40% effacement, -3 station with low concern for preterm labor based on assessment. She was overall well appearing with ongoing mild-range blood pressures. A few hours later, the patient acutely developed headache, agitation, tremors, diaphoresis, and persistent tachycardia ranging from 120 to 130 bpm. The patient also had severe hypertension with markedly wide pulse pressures (e.g., 182/85, 175/76) that were refractory to multiple doses of IV antihypertensives. She was transferred to the intensive care unit (ICU) due to great clinical concern for thyroid storm. MMI was discontinued, and the patient was initiated on standard thyroid storm therapy ( Fig. 1 ), including esmolol infusion, PTU, hydrocortisone, cholestyramine, and potassium iodide.

On hospital day 3, while in the ICU for thyroid storm treatment, the patient developed worsening lower abdominal contractions. Cervical exam showed progression to 2 cm, raising concern for preterm labor in the setting of critical maternal illness. Neonatology colleagues were consulted to discuss periviability and neonatal resuscitation preferences, and betamethasone was administered as she opted to pursue a trial of neonatal resuscitation in the event of delivery. A ropivacaine epidural was placed overnight for pain control with a stabilizing cervical exam. Additionally, plasmapheresis was initiated as adjunctive therapy for refractory thyroid storm.

On hospital day 4, the epidural was paused, and shortly thereafter, she reported worsening pelvic pressure and urge to push. Cervical exam confirmed complete dilation with the fetal head of Twin A visible in the vagina on transabdominal ultrasound. With obstetric and neonatology teams present, she delivered Twin A spontaneously (535 g), followed by an uncomplicated breech delivery of Twin B (560 g); both were liveborn, intubated at birth, and transferred to the neonatal intensive care unit (NICU).

**Fig. 2:**
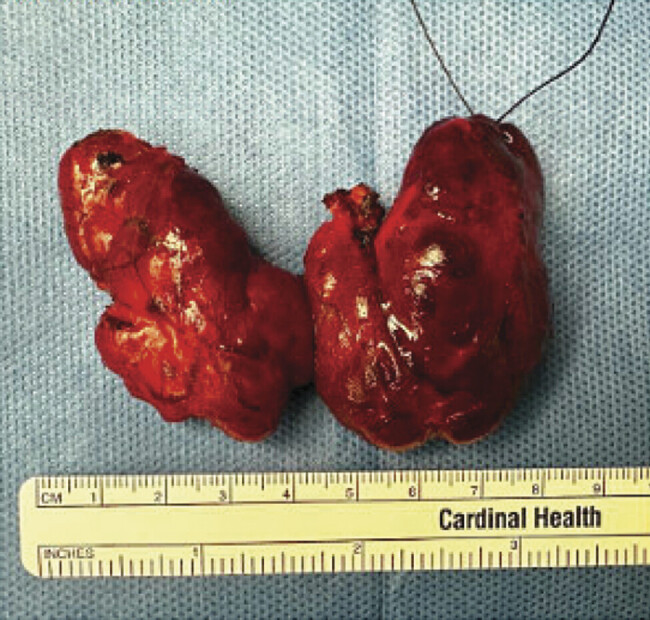
Gross specimen of the thyroid gland following total thyroidectomy. The right lobe measured 5.3 cm × 3.2 cm × 2.6 cm, the left lobe 5.8 cm × 3.0 cm × 2.0 cm, and the isthmus 2.0 cm × 1.5 cm × 0.5 cm. Subsequent pathological evaluation confirmed diffuse follicular hyperplasia.

The patient met all postpartum milestones by postpartum day 2; however, she remained clinically hyperthyroid despite near-maximal medical therapy for thyroid storm and several sessions of plasmapheresis ( Table 1 ). She ultimately underwent definitive treatment with a total thyroidectomy on hospital day 12 (postpartum day 8). Fig. 2 shows the gross thyroid specimen, and subsequent pathological evaluation demonstrated diffuse follicular hyperplasia.

**Table 1:** Timeline of clinical course

Hospital day	Level of care	BP range	HR range	Maximum oxygen requirement	Antihypertensive medication	Free T4 (ng/dL) a	Key events/interventions
1	Antepartum	Min: 144/67 Max: 161/70	Avg: 107 Min:102 Max:114	–	Oral labetalol	–	Admitted to antepartum service for blood pressure and laboratory monitoring
2	ICU	Min: 131/66 Max: 182/85	Avg: 118.9 Min: 99 Max: 134	–	Oral and intravenous labetalol → Esmolol and nicardipine infusion	4.62	Initiated empiric magnesium for eclampsia prophylaxis Initiated antithyroid medications for thyroid storm
3	ICU	Min: 118/67 Max: 144/67	Avg: 109 Min: 99 Max: 118	4 L nasal cannula	Esmolol and nicardipine infusion	3.97	Plasmapheresis #1 Discontinued magnesium for eclampsia prophylaxis Betamethasone is administered for fetal pulmonary advancement
4	ICU	Min: 118/67 Max: 149/65	Avg: 103.3 Min: 93 Max: 114	4 L nasal cannula	Esmolol and nicardipine infusion, Oral propranolol	4.21	Preterm delivery of twins Plasmapheresis #2 Chest X-ray with pulmonary edema
5	ICU	Min: 127/49 Max: 176/67	Avg: 105.7 Min: 86 Max: 117	3 L nasal cannula	Esmolol and nicardipine infusion, Oral propranolol	4.39	Plasmapheresis #3 Endocrine surgery was consulted for consideration of thyroidectomy
6	ICU	Min: 130/55 Max: 147/63	Avg: 97.4 Min: 90 Max: 116	6 L nasal cannula	Nicardipine infusion, Oral propranolol, and candesartan	2.15	Plasmapheresis #4
7	ICU	Min: 147/52 Max: 147/52	Avg: 98.1 Min: 88 Max: 116	6 L nasal cannula	Nicardipine infusion, Oral propranolol, candesartan, and amlodipine	2.10	–
8	ICU	Min: 112/56 Max: 122/51	Avg: 92.7 Min: 84 Max: 103	2 L nasal cannula	Nicardipine infusion, Oral propranolol, candesartan, and amlodipine	1.46	Plasmapheresis #5
9	ICU	Min: 126/59 Max: 157/58	Avg: 87.4 Min: 77 Max: 97	–	Nicardipine infusion, Oral propranolol, candesartan, and amlodipine	1.37	–
10	ICU	Min: 102/57 Max: 155/67	Avg: 87.2 Min: 76 Max: 97	–	Nicardipine infusion, Oral propranolol	1.10 b	–
11	ICU	Min: 97/80 Max: 150/79	Avg: 81.8 Min: 70 Max: 94	–	Nicardipine infusion, Oral, propranolol	1.16 b	Plasmapheresis #6
12	General medicine service	Min: 88/61 Max: 146/80	Avg: 74 Min: 65 Max: 99	–	–	1.09 b	Plasmapheresis #7 Total thyroidectomy Discontinued antithyroid medications postoperatively
13	General medicine service	Min: 142/86 Max: 157/95	Avg: 91 Min: 69 Max: 109	–	–	0.92 b	–
14	General medicine service	Min: 124/77 Max: 147/89	Avg: 95 Min: 84 Max: 109	–	Amlodipine 10 mg, atenolol 25 mg daily	0.97 b	–
15	General medicine service	Min: 127/81 Max: 147/84	Avg: 85 Min: 68 Max: 103	–	Amlodipine 10 mg, atenolol 25 mg daily	1.13 b	Initiated levothyroxine
16	General medicine service	Min: 132/77 Max: 141/84	75	–	Amlodipine 10 mg, atenolol 25 mg daily	0.76 b	Discharged home

a Normal range for free T4 is 0.52 to 1.21 ng/dL. Target free T4 in pregnancy: high-normal or slightly elevated value, independent of TSH levels. 1

b Within normal limits.

She was discharged on hospital day 16 with oral antihypertensives and levothyroxine for management of postsurgical hypothyroidism. Her twins remained in the NICU for extreme prematurity and treatment of neonatal Graves' disease.

## Discussion

This case illustrates several important clinical implications for managing severe endocrinopathies in pregnancy. First, thyroid storm can closely mimic hypertensive disorders of pregnancy, presenting with severe hypertension, neurologic symptoms, hepatic and renal dysfunction (elevated transaminases and creatinine), and in severe cases, pulmonary edema—all features that overlap with preeclampsia with severe features. 5 6 Given that thyroid storm is a clinical diagnosis, clues like our patient's hypertension with pulse pressures > 50 mmHg and associated tachycardia were critical for recognition. Obtaining a catheterized urine specimen was also helpful in excluding superimposed preeclampsia. Finally, considering other clinical clues, such as gestational age, is essential, as preeclampsia before viability is rare even in higher-risk multiple gestations. 1

The American College of Obstetricians and Gynecologists (ACOG) recommends treating thyroid storm in pregnancy with antithyroid drugs such as PTU, iodine, corticosteroids (dexamethasone or hydrocortisone), and a beta-blocker. 1 ACOG also notes that if fetal status is unstable during thyroid storm, stabilization can often be achieved with appropriate maternal treatment. This parallels other critical illnesses in pregnancy, such as sepsis and diabetic ketoacidosis, where maternal stabilization can improve fetal status. Importantly, avoiding delivery during active thyroid storm is recommended when possible.

In most cases, patients with thyroid storm begin to show clinical improvement within 24 to 48 hours of initiating standard therapy. Plasmapheresis is typically reserved for cases that are refractory to medical management, with prior reports suggesting an average of four to six sessions may be required to achieve clinical improvement. 7 However, because its effects are transient, plasmapheresis is often used as an adjunct to standard therapy or as a bridge to definitive surgical treatment. 6

A key point illustrated by this case is that the degree of thyroid function test abnormality does not always correlate with clinical severity. 3 Notably, our patient's outpatient studies were more abnormal than those at the onset of thyroid storm. Additionally, despite multiple inpatient plasmapheresis sessions and improving laboratory values, her clinical status failed to improve, prompting surgical consultation, ultimately thyroidectomy. This underscores that while laboratory values provide useful context, clinical stabilization remains the cornerstone of management.

This case also highlights the importance of identifying stressors in pregnancy that may precipitate thyroid storm, such as hypertensive crises or infection. While uncontrolled hyperthyroidism, as in our patient, is a well-recognized risk factor, it is not itself a trigger. 1 6 The precipitant was unclear, as thyroid storm may have contributed to the onset of labor, while labor could also have precipitated thyroid storm, creating a cycle in which each process amplified the other. Reported precipitants in other cases include acute myocarditis 8 or cesarean delivery, 9 though the antecedent stressor is sometimes less apparent.

Finally, our case supports prior literature demonstrating that critical maternal illness can precipitate preterm birth. 10 With additional risk factors of multiple gestation and a short cervix, this patient was at even greater risk of spontaneous preterm delivery. Notably, there is a reported case of thyroid storm resulting in placental abruption and preterm delivery at 29 weeks' gestation 11 and prior literature indicates that pregnant patients with uncontrolled hyperthyroidism have 9.24-fold increased odds of delivering low birth weight infants compared to controls, 12 supporting the idea that hyperthyroidism itself may contribute to preterm birth.

In summary, we reported a case of thyroid storm in a twin pregnancy presenting as severe hypertension and preterm labor. This case reinforces the importance of early recognition and timely, evidence-based management of thyroid storm in pregnancy, as maternal stabilization is key to optimizing both maternal and fetal outcomes.

## References

[1] Thyroid Disease in Pregnancy Thyroid disease in pregnancy: ACOG Practice Bulletin, Number 223 Obstet Gynecol 2020 135 06 e261 e274 32443080 10.1097/AOG.0000000000003893

[2] Waltman P A Brewer J M Lobert S Thyroid storm during pregnancy. A medical emergency Crit Care Nurse 2004 24 02 74 79 15098313

[3] Cooper D S Laurberg P Hyperthyroidism in pregnancy Lancet Diabetes Endocrinol 2013 1 03 238 249 24622372 10.1016/S2213-8587(13)70086-X

[4] Burch H B Wartofsky L Life-threatening thyrotoxicosis. Thyroid storm Endocrinol Metab Clin North Am 1993 22 02 263 277 8325286

[5] Chen Y H Liao C P Lu C W Lin T Y Chang Y Y Thyroid storm superimposed on gestational hypertension: a case report and review of literature Medicina (Kaunas) 2022 58 03 450 35334626 10.3390/medicina58030450 PMC8951575

[6] Vadini V Vasistha P Shalit A Maraka S Thyroid storm in pregnancy: a review Thyroid Res 2024 17 01 2 38229163 10.1186/s13044-024-00190-y PMC10792856

[7] De Almeida R McCalmon S Cabandugama P K Clinical review and update on the management of thyroid storm Mo Med 2022 119 04 366 371 36118802 PMC9462913

[8] Maltese V Gatta E Silvestrini I An Unusual and severe thyrotoxicosis in a twin pregnancy: fortune favors the brave Case Rep Endocrinol 2025 2025 6.298137E6 10.1155/crie/6298137 PMC11748746 39838969

[9] Ma Y Li H Liu J Lin X Liu H Impending thyroid storm in a pregnant woman with undiagnosed hyperthyroidism: a case report and literature review Medicine (Baltimore) 2018 97 03 e9606 29504986 10.1097/MD.0000000000009606 PMC5779755

[10] for PregCOV-19 Living Systematic Review Consortium Allotey J Stallings E Bonet M Clinical manifestations, risk factors, and maternal and perinatal outcomes of coronavirus disease 2019 in pregnancy: living systematic review and meta-analysis BMJ 2020 370 m3320 32873575 10.1136/bmj.m3320 PMC7459193

[11] Lane A S Tarvade S Thyroid storm causing placental abruption: cardiovascular and management complications for the Intensivist J Intensive Care Soc 2015 16 03 247 252 28979421 10.1177/1751143714559910 PMC5606429

[12] Millar L K Wing D A Leung A S Koonings P P Montoro M N Mestman J H Low birth weight and preeclampsia in pregnancies complicated by hyperthyroidism Obstet Gynecol 1994 84 06 946 949 7970474

